# Follow-up of complex hernia repair with intraoperative fascial traction

**DOI:** 10.1007/s10029-025-03297-9

**Published:** 2025-05-02

**Authors:** Guido Woeste, Sandrina Dascalescu, Felix Wegner, Hansjörg Meier, Nihad Sardoschau, Adrien Kiehle, Halil Dag, Zaid Malaibari, Henning Niebuhr

**Affiliations:** 1 Goethe University Frankfurt, Faculty of Medicine, Frankfurt A.M., Germany; 2 Agaplesion Elisabethenstift Darmstadt, Darmstadt, Germany; 3 Sana-Hospital Düsseldorf-Benrath, Düsseldorf, Germany; 4 Agaplesion Bethesda Hospital Bergedorf, Bergedorf, Germany; 5 LaSar Saarbrücken, Saarbrücken, Germany; 6 St. Elisabeth Hospital Leipzig, Leipzig, Germany; 7 Hamburg Hernia Center, Hamburg, Germany; 8 University of Tabuk, Faculty of Medicine, Department of Surgery, Tabuk, Saudi Arabia

**Keywords:** Fascial traction, Fasciotens, Follow-up, IFT, Ventral hernia repair, Complex hernia

## Abstract

**Background:**

Different techniques for complex abdominal wall repair are utilised including intraoperative fascial traction (IFT) as the latest development. Despite increasing case numbers for IFT across Europe, long-term data especially on recurrence rates are not available yet.

**Methods:**

Follow-up data from five different German hernia centers between 12/2019 and 9/2023 were assessed. All patients received Rives-Stoppa repair (RSR) and IFT intraoperatively with an additional transverse abdominis muscle release (TAR) in some cases. 30-day postoperative outcome data were retrospectively collected Standardized follow-up was performed after a minimum of 3 months including clinical examination and standardized ultrasound.

**Results:**

A total of 100 patients were included in the study. The mean age was 60.7 ± 14.3 years; the mean BMI was 31.3 ± 7.3 kg/m² with a mean follow-up of 19.7 ± 10.7 months. The mean defect width was 15.8 ± 5.2 cm. In 94% of the patients complete fascial closure was achieved; in 28% an additional TAR procedure was necessary During follow-up, 2 recurrences were found. The surgical site occurrence (SSO) rate was 33% including mainly seromas (54.5%) and surgical site infections (SSI) of 9% Comparing the groups of IFT + TAR and IFT + RSR a significantly higher incidence of SSO was found in the TAR group (50% vs. 26.4%, *p*<0.01).

**Conclusions:**

This study, which is the first long-term follow-up, shows very promising results of the innovative IFT technique in terms of closure rate, wound morbidity, and recurrence rate.

## Introduction

Abdominal wall defects such as primary or secondary hernias represent one of the most common procedures in general surgery. Because of different techniques used and propagated by different surgeons, the repair is not yet really standardized [1]. Furthermore, the anatomical reconstruction of major defects with a width of almost or even more than 10 cm requires technically and anatomically challenging procedures, especially after previous failed attempts. The literature clearly shows that bridging techniques are functionally inferior to exact anatomical reconstruction [2]. Furthermore, these procedures are associated with a high number of wound complications with the common sequelae of mesh infection [3]. Myofascial advancement in order to close the defect can be achieved by different forms of component separation techniques [4–6]. Despite the development of sophisticated open and endoscopic approaches to preserve the blood supply of the skin flaps the anterior component separation is complicated by a high frequency of wound complications such as infection and seroma [7, 8]. The posterior approach in its mostly used modification of the transversus abdominis muscle release (TAR) seems to reveal better results in terms of reduced surgical site occurrences (SSO) [9]. However, according to a comprehensive systematic review, neither the open nor the endoscopic approach has been clearly proven superior to the anterior approach, despite some minor supporting evidence in the literature [5, 10, 11]. The robotic approach may show better results, however the technique is not generally available and cannot be used in all cases of secondary large abdominal wall defects [12, 13]. However, it should be kept in mind that these reviews suffer from generally poor quality of the underlying data.

Fascial traction which is used in pediatric surgery for decades was first described in 2017 by D. Eucker et al. in a series of desperate cases [14, 15]. That technique, however, was not standardized at all concerning the force of traction. Since 2021, the fasciotens^®^hernia (Fasciotens GmbH, Essen, Germany) device for Intraoperative Fascial Traction (IFT) is available in the European market and beyond and is being increasingly used. Some minor series with up to 50 patients and one large series including 143 patients have been published showing the effect of standardized fascial traction on myofascial advancement [16–18]. The frequency of component separation techniques could be reduced at least in cases with midline defects without any previous stoma, trocar, and drainage sites, as well as the rate of midline closure proved to be exceptionally high.

TAR protagonists generally suppose that the use of very large meshes, which is only possible after TAR, is essential for a safe and long-lasting repair. However, no scientific data supports this point of view. On the other hand, although lacking scientific data, the scar formation induced by these very large meshes may interfere much more with the abdominal wall function compared to a simple retrorectus augmentation of the midline.

The present study summarizes peri- and postoperative data of 100 consecutive patients with W3 hernias of the midline treated in 5 different specialized Hernia centers in Germany. The data were collected using standardized documentation. The examination comprises well-defined clinical data and a standardized ultrasound protocol at different time points with a minimum follow-up time of 3 months. The data analysis focused on recurrence rate by clinical and standardized ultrasound examination, rate of anterior rectus sheath closure, the necessity of additional component separation procedures and perioperative complications.

## Materials and methods

### Patient population

Patients from five different high volume certified hernia centers in Germany treated between August 2019 and September 2023 were included. Between August 2019 and April 2021, the fasciotens^®^ Abdomen device was used for IFT, thereafter the fasciotens^®^ Hernia system was applied. All patients were treated for midline incisional hernia repair including IFT which was previously described [16, 17]. For the study presented, only cases in which anterior fascial closure at low tension was not possible without using IFT were included for data analysis.

Postoperative routine follow-up was realised within 30 days after surgery. Afterward, patients are encouraged to undergo yearly follow-ups. For this follow-up, study patients were individually invited by each certified hernia center for an additional outpatient consultation.

### Surgical procedure

**Fig. 1:**
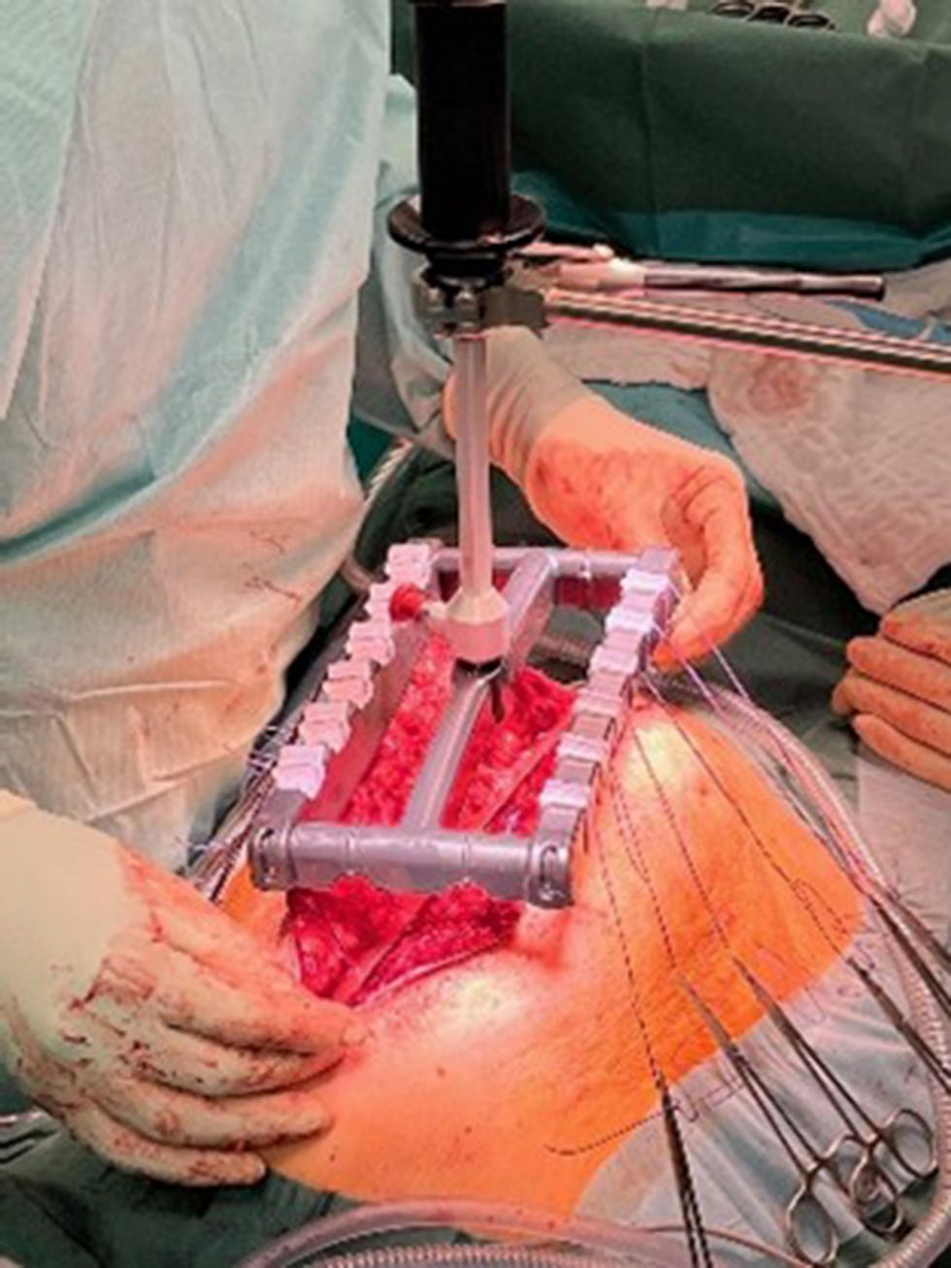
Intraoperative impressions of Intraoperative Fascial Traction (IFT)– courtesy of Guido Woeste, Agaplesion Hospital Darmstadt, Germany and Hansjörg Meier, Sana-Hospital Düsseldorf-Benrath, Germany

All patients received a CT scan or MRI for preoperative planning. In 87% of cases, the patients were treated with Botulinum Toxin A (BTA) preoperatively. The injected units and protocols differed by each hernia center. Intraoperatively, an attempt was made to leave the hernia sac intact (if possible) or the peritoneal flap technique according to Malik et al. was performed [19]. Restrained adhesiolysis and retrorectus dissection as described by Rives and Stoppa was performed. In some cases, an additional TAR was carried out. Main causes were a former stoma side, an additional lateral defect or unavailability of a peritoneal flap for posterior sheath closure (especially after incisional hernia following laporostomy). The posterior rectus sheaths were either closed directly or by using peritoneal flap. All patients but one received mesh augmentation in the sublay (retrorectus) position using different types of meshes as described in Fig. 3. IFT was used if a low-tension fascial closure was not achievable for the anterior rectus sheaths. All centers are using a specially designed medical device from fasciotens (Fasciotens Gmbh, Germany) for controlled IFT, which is a combination of a reusable arm attached to the OR table and a single-use traction unit. The cost for the single-use part is comparable to a small to medium sized biological mesh. An increasing number of German private and public health insurance companies are already covering the patient-related cost. To carry out IFT, 12 polyfilamentel surgical sutures (Vicryl^TM^Plus, USP 2, Ethicon^®^, USA) (6 U-sutures distributed equally on each side) are sutured about 1 cm lateral to the margin of the anterior rectus sheaths on each side with a stitch length of 2–3 cm. The sutures are then aligned crosswise and clamped in the suture retention frame of the IFT device [20]. We used a traction force between approx. 14–20 kg in this cohort (Fig. 1). Traction was carried out for 30 min. Generally, the handling of the device is quite intuitive and the surgeons in our group felt comfortable to use the technique after two proctored cases on average. If a low-tension closure of the anterior sheaths was not feasible after IFT, additional bridging using either part of the hernia sac (peritoneal flap technique) or a further mesh was performed.

### Data collection

During outpatient consultation written consent for data collection and processing as part of the study was given. Prospective data on 30 days postoperative outcome were retrospectively collected individually by each certified hernia center. The minimum follow-up period was 3 months for long-term clinical outcomes. During outpatient consultation, each patient underwent a physical examination of the abdomen by one of the specialized abdominal wall reconstruction (AWR) surgeons in each hospital. Additionally, a standardized dynamic abdominal wall ultrasound (DAWUS) was performed. We established this examination according to the dynamic inguinal ultrasound (DIUS) of groin hernias [21]. The following parameters were assessed using the DAWUS protocol:

- The midline.
- The posterior and anterior rectus sheaths.
- The distance of the rectus muscle 5 cm and 2 cm cranial the umbilicus as well as 5 cm caudal the umbilicus.
- Possible fluid collections.
- Positioning of the mesh if visible.

In case of the possibility of hernia recurrence patients were referred for a CT scan. Each patient was assessed for patient-reported outcomes (PRO) using the HerQles hernia-related quality of life (QOL) survey [4]. Data analysis was carried out using Minitab^®^ Statistical Software Version 21.3 (Minitab, LLC; State College, Pennsylvania, USA). Differences between groups were tested using Chi-square or Fisher’s exact test. The level of significance was determined with α = 0.05.

### Clinical outcomes

Hernia recurrence as the primary clinical outcome was assessed by physical examination and DAWUS. Patient-related outcomes (e.g., BMI, age), surgery-related outcomes, and 30-day postoperative findings were collected (e.g., length of stay, surgical site occurrence) retrospectively. For standardization, we used the classification for postoperative complications which was proposed by Haskins et al. [22]. Using a standardized protocol for DAWUS abdominal wall properties were collected. The protocol can be found in the supplementary data. Data sets were stored by each hernia center in an individual Excel sheet.

## Results

A total of 101 patients were scheduled by the hernia centers for a standardized follow-up. 1 patient was excluded from the evaluation due to too short a follow-up period (less than 3 months). The hernia centers included the following number of patients:

- Hamburg Hernia Center 62 patients.
- Sana-Hospital Dusseldorf-Benrath 13 patients.
- Agaplesion Hospital Darmstadt 11 patients.
- Lasar Saarbrücken 9 patients.
- St. Elisabeth Hospital Leipzig 5 patients.

**Fig. 2:**
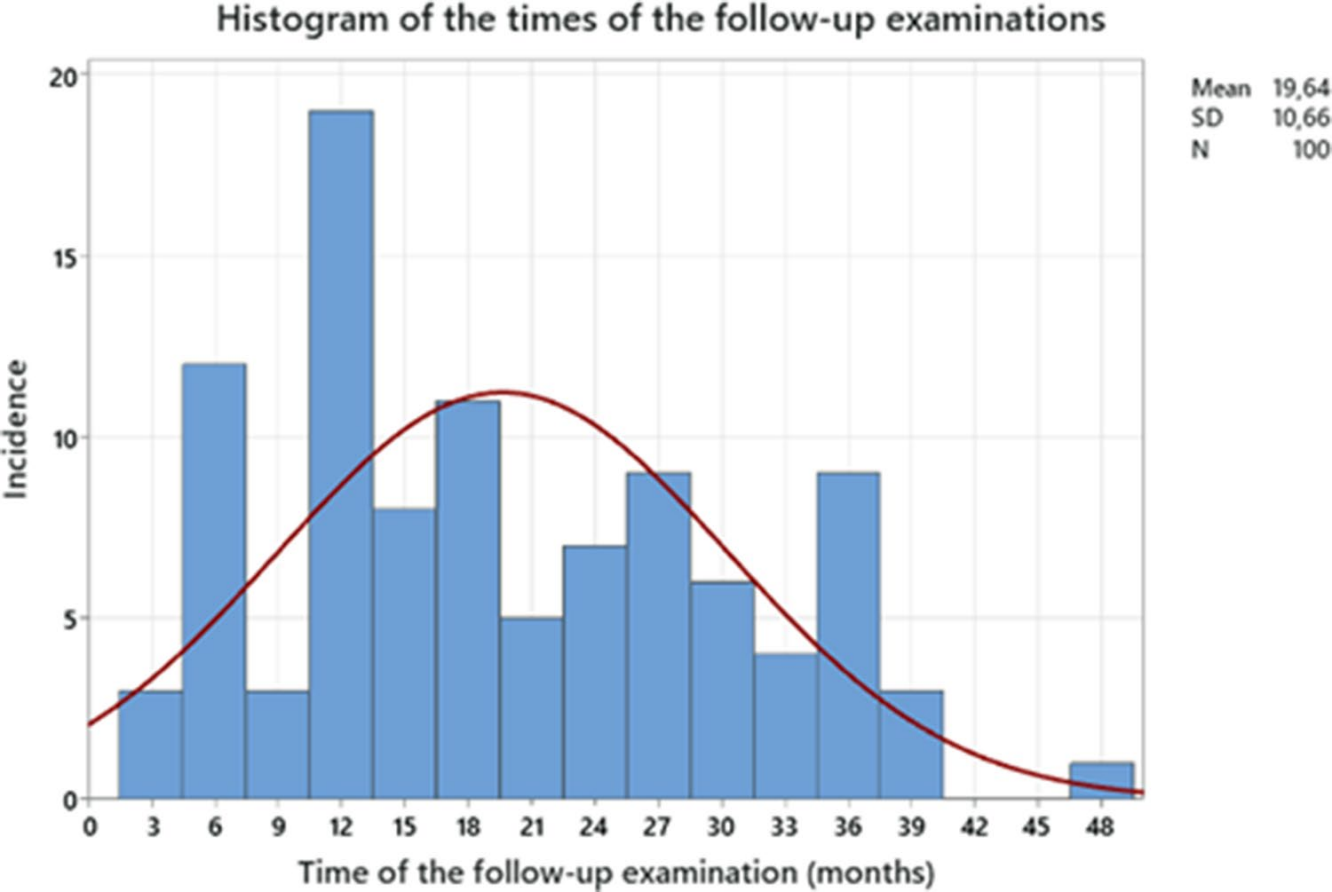
Distribution of follow-up time periods

All patients had an incisional or recurrent incisional midline hernia. The patient collective was nearly balanced between males and females with a slide domination of males (55%). Mean age was 60.7 ± 14.3 (mean and standard error of the mean (SEM)). Mean BMI was 31.3 ± 7.3 (Mean ± SEM). The mean follow-up time was 19.6 ± 10.7 months (mean and SEM). 76% of all patients had a follow-up time of 12 months or more. Patient characteristics are summarised in Table 1. The distribution of the different periods is visualized in Fig. 2.

**Table 1:** Patient characteristics; SEM - standard error of the mean, IQR– interquartile range

**Patient characteristics** (***n* = 100**)	
**Gender (male/female)**	55% / 45%
**Age (years)**	
*Mean value ± SEM*	60.7 ± 14.3
*Median*,* IQR*	60 (52.8–70.0)
Body mass index (kg/m2)	
*Mean value ± SEM*	31.3 ± 7.3
*Median*,* IQR*	30.0 (25.5–36.1)
Year of Operation	
*2019*	1
*2020*	16
*2021*	27
*2022*	39
*2023*	17
Follow-up time (months)	
*Mean value ± SEM*	19.6 ± 10.7
*Median*,* IQR*	17 (12–28)

The intraoperative details are summarized in Table 2. The defect width was 15.8 ± 5.2 cm (mean and SEM). 87% of all patients were pretreated with BTA before the hernia repair. An additional TAR was performed in 28% of all cases. Overall, the closure rate for the anterior rectus sheaths was 94%. Interestingly the defect surface had no significant influence on performing an additional TAR (*p* = 0.199; α = 0.05). Neither the pretreatment with BTA nor performing a TAR had a significant influence on the closure rate (*p* = 1 respectively *p* = 0.216; α = 0.05). An intraoperative bowel lesion occurred in two cases.

**Table 2:** Intraoperative Details; SEM - standard error of the mean, TAR– transverse abdominis release

**Operative Details** (***n* = 100)**	
**Defect width (cm)**	
*Mean value ± SEM*	15.8 ± 5.2
*Median*,* Range*	15 (8–44)
**Defect surface (cm²)**	
*Mean value ± SEM*	341.3 ± 246.3
*Median*,* Range*	288 (66–1892)
BTA	87 (87%)
TAR	28 (28%)
Bridging	6 (6%)
Mesh size	
*Length (cm)*	
*Mean value ± SEM*	31.6 ± 6.6
*Median*,* Range*	30 (15–45)
*Width (cm)*	
*Mean value ± SEM*	22.6 ± 8.9
*Median*,* Range*	20 (10–60)
Mesh surface (cm²)	
*Mean value ± SEM*	727.2 ± 404.6
*Median*,* Range*	600 (0–2700)
Intraoperative Complications	2 (2%)

**Fig. 3:**
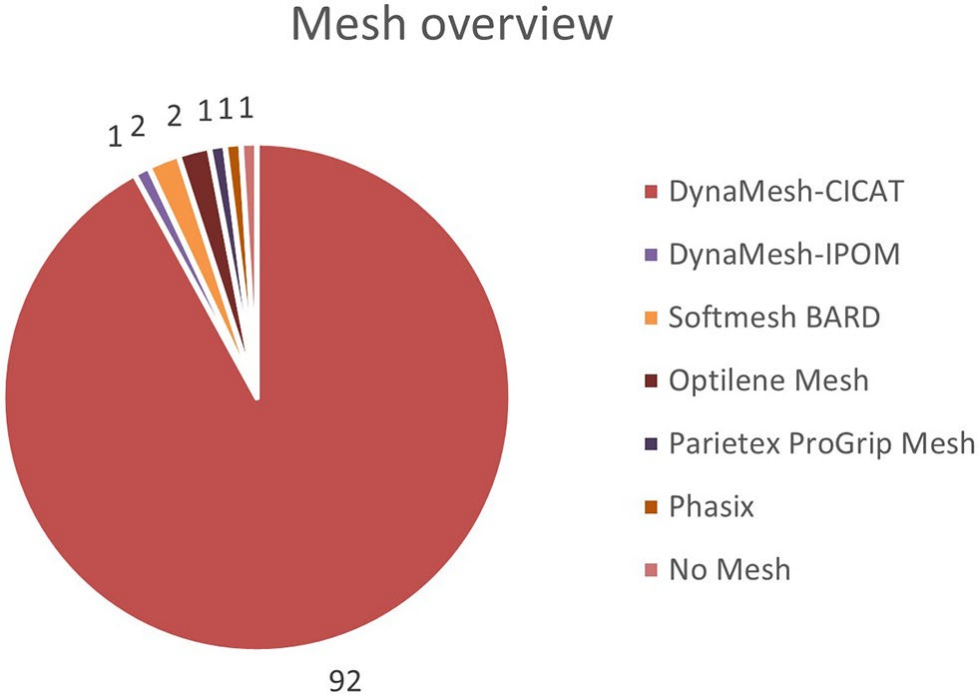
Overview of Mesh used for retrorectus augmentation

All but one case had a sublay mesh augmentation using a synthetic mesh. The mean mesh surface was 727.2 cm² ± 404.6 (mean and SEM). The mean defect and mesh surface in the group treated only with Rives-Stoppa and IFT was 332.3 cm² and 654.5 cm² respectively, leading to a defect-to mesh ratio of 1:1.97. Hence, the mean defect and mesh surface in the group treated by additional TAR were 364.5 cm² and 914.0 cm² respectively, leading to a defect-to-mesh ratio of 1:2.51. However, the mesh width was significantly higher when an additional TAR was performed (*p* = 0.012; α = 0.05). In one case, the surgeon decided against a mesh augmentation due to the young age of the patient. In the vast majority of cases (92.9%) a DynaMesh^®^-CICAT (FEG mbh, Aachen, Germany) was used for mesh augmentation. The meshes used for augmentation are shown in Fig. 3.

Follow-up was carried out during a scheduled outpatient monitoring. All patients underwent thorough clinical examination of the abdominal wall including Valsalva manoeuvre. Two patients showed clinical and sonographic signs of a recurrence in form of a mesh border hernia. One patient had an additional TAR in the initial operation, the other one was treated only by BTA, Rives-Stoppa and IFT. The patients were reoperated during the follow-up period and had no recurrence at the latest appointment. The clinical examination was followed by a standardized ultrasound (DAWUS) of the abdominal wall. The results can be found in Table 3. Two patients were excluded from the ultrasound examination since they received CT scans as radiological follow-up. One patient showed signs of a subcutaneous fluid collection and received a CT scan 5 months postoperatively. It showed a large seroma which was treated by surgical excision followed by negative pressure wound therapy (NPWT). Another patient showed signs of an ileus 15 months after hernia repair. The CT scan revealed an impaired transit due to adhesions and the patient was treated surgically. Both patients did not show any signs of recurrence in either the CT scan or intraoperatively.

**Table 3:** Results of standardized ultrasound examination

Sonography protocol evaluation (*n* = 98)	
Identification Linea alba	88 (89.8%)
Linea alba can be displayed continuously?	78 (79.6%)
Can the anterior sheath of the rectus be visualized?	92 (93.9%)
Can the posterior sheath of the rectus be visualized?	55 (56.1%)
Identification rectus muscles?	92 (93.9%)
Identification mesh	74 (75.5%)
Identification fluid collection (*n* = 100)	13 (13.0%)
Surgical Intervention due to fluid collection	2 (2.0%)
Recurrence rate (*n* = 100)	2 (2.0%)
Distance between rectus muscles (*n* = 95)	
*≤ 2.0 cm*	42
*> 2.0 cm*	53

Linea alba was identifiable in 89.8% of all cases whereas both rectus muscles were identifiable in 93.9% and the anterior rectus sheath also in 93.9%. Naturally, the posterior rectus sheath were only visible in 56.1% of all cases mainly due to ultrasound interference of the synthetic mesh. The distance between the medial borders of the rectus muscle was measured at three different points on the abdominal wall (5 and 2 cm cranial of the umbilicus and 5 cm caudal of the umbilicus). Measurement was possible in 95 of the cases and 42 patients (44.2%) had a mean distance less than or equal to 2 cm.

For the 30-day postoperative outcome, a retrospective analysis for all cases was carried out individually by each participating hernia center. For the definition of SSO and Surgical Site Infections (SSI) as part of SSO, the definition by Haskins et al. was used [22]. A total of 33 patients had a postoperative SSO. 16 of them needed an intervention. The majority of SSOs were seromas (54.5%). However, the rate of SSIs and SSOs differed between the patients who only had IFT and patients who were treated with IFT and TAR (10.7% vs. 8.3% and 50% vs. 26.4% respectively). A summary of the postoperative outcome is shown in Table 4.

**Table 4:** 30-day postoperative outcome

Postoperative Outcome (*n* = 100)	
Postoperative Surgical Site Occurrences	33 (33%)
Postoperative Surgical Site Infections	9 (9%)
Need for intervention	16 (16%)
Postoperative Non-Surgical Complications	5 (5%)
Length of stay (days)	
*Mean value ± SEM*	8.8 ± 11.8
*Median*,* Range*	6 (2–103)

The occurrence of a postoperative SSO was significantly higher if an additional TAR was performed (*p* = 0.024, α = 0.05) as well as the development of a seroma (*p* = 0.008, α = 0.05).

**Fig. 4:**
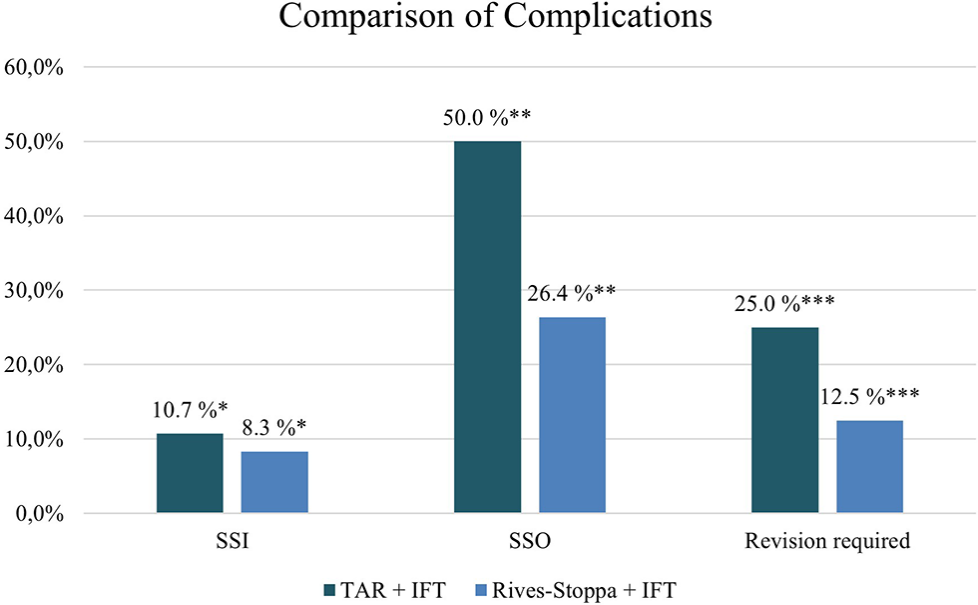
Comparison of postoperative complications and revisions for patients treated either with transverse abdominis muscle release (TAR) and intraoperative fascial traction (IFT) or Rives-Stoppa and IFT; SSO– Surgical Site Occurrence, SSI– Surgical Site Infection; * not significant (*p* = 0.709, α = 0.05),** (*p* = 0.024, α = 0.05),*** (*p* = 0.126, α = 0.05)

**Fig. 5:**
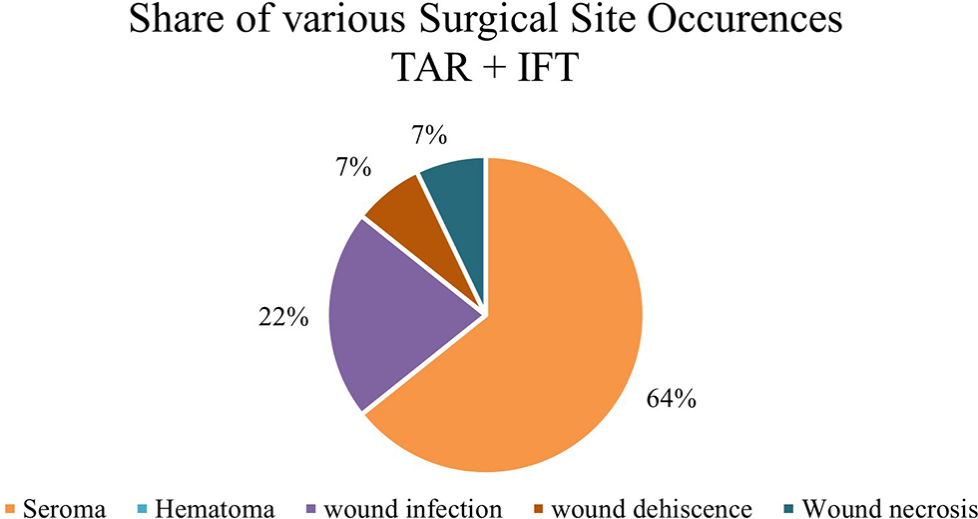
Various SSOs for patients treated with transverse abdominis release (TAR) and intraoperative fascial traction (IFT)– *N* = 14

**Fig. 6:**
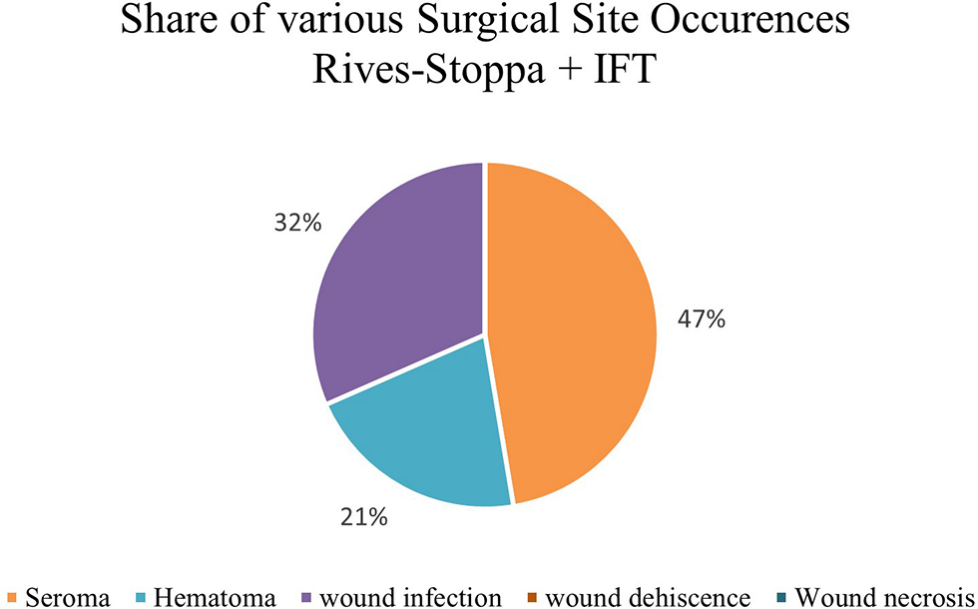
Various SSOs for patients treated with Rives-Stoppa and intraoperative fascial traction (IFT)– *N* = 19

Figure 4 shows the comparison of postoperative complications and revisions for patients treated either with transverse TAR and IFT or just Rives-Stoppa and IFT. Figures 5 and 6 illustrate the various SSOs that occurred in patients with and without additional TAR.

Interestingly, the number of cases in need for a revision was higher if an additional TAR was performed. However, the need for revision due to SSO was not significantly higher in the TAR group (*p* = 0.126, α = 0.05). Those patients (*N* = 16), needing a revision, were either treated with a drain, wound revision, or NPWT. If patients had an additional TAR the percentage of wound revision or NPWT was higher than treatment with Rives-Stoppa and IFT alone (17.9% and 8.3% respectively.). Five patients developed a non-surgical postoperative complication. The mean length of stay (LOS) was 8.8 ± 11.8 (mean and SEM) in this patient cohort. Interestingly, the mean LOS when IFT and TAR was performed was 11.5 vs. 7.8 days if only IFT and Rives-Stoppa was carried out.

All patients completed a HerQles questionnaire during the follow-up appointment. Naturally, this means that no comparison can be made between the situation before and after hernia surgery at this point. The HerQles Quality of Life score consists of 12 questions concerning the abdominal wall function in daily life. Questions can be answered by numbers from 1 to 6 where 1 is complete disapproval and 6 is complete approval. After all answers have been added up a score is calculated using the formula 120 - ((20/12) x (Total of questions 1–12)). In our patient cohort, the mean HerQles Summary Score was 68.47 ± 16.3 (mean and SEM). Summarized in Table 5 are the mean values and SEM of all 12 questions.

**Table 5:** HerQles score with mean and SEM for all questions; SEM - standard error of the mean

HerQles (*n* = 100)	
Question	Mean ± SEM
1) My abdominal wall has a huge impact on my health	3.95 ± 1.45
2) My abdominal wall causes me physical pain	2.60 ± 1.11
3) My abdominal wall interferes when I perform strenuous activities, e.g. heavy lifting	2.88 ± 1.27
4) My abdominal wall interferes when I perform moderate activities, e.g. bowling, bending over	2.54 ± 1.13
5) My abdominal wall interferes when I walk of climb stairs	2.34 ± 1.08
6) My abdominal wall interferes when I dress myself, take showers and cook	2.31 ± 1.04
7) My abdominal wall interferes with my sexual activity	2.39 ± 1.10
8) I often stay at home because of my abdominal wall	2.14 ± 1.00
9) I accomplish less at home because of my abdominal wall	2.24 ± 1.09
10) I accomplish less at work because of my abdominal wall	2.40 ± 1.21
11) My abdominal wall affects how I feel every day	2.74 ± 1.29
12) I often feel blue because of my abdominal wall	2.41 ± 1.19

## Discussion

IFT used for complex hernia repair applies controlled traction forces to the anterior rectus sheaths to elongate the myofascial structures. The primary goal is to facilitate and achieve a low-tension closure of the anterior rectus sheath, which is one of the key principles of abdominal wall hernia reconstruction and strongly recommended by the EHS [20]. Currently, the use of IFT is suggested for abdominal wall reconstruction of hernias with large gaps of 10 cm width and above (EHS W3 hernias) and if fascial closure of the anterior sheaths is deemed impossible. Hence, IFT is used on demand and the decision is normally made intraoperatively. In case that closure of the anterior rectus sheaths after Rives-Stoppa dissection is possible with moderate tension, closure without utilising IFT can be performed. However, if there is still too much tension to close the anterior fascia directly, IFT is applied. In our experience, in some cases rather large defects can be closed easily while sometimes even W2 hernias require IFT due to low abdominal wall compliance. Therefore, we believe that the technique should be available in hospitals that regularly treat complex hernia cases.

Since its introduction IFT has had an internationally growing number of applications. Recent publications on IFT have focused on intraoperative findings and short-term postoperative outcomes [15–18, 23–25]. However, long-term outcomes, especially recurrence rates, are crucial in complex abdominal wall repair as they determine whether a patient’s quality of life can improve. Therefore, we initiated a follow-up trial (minimum follow-up 3 months) in our patient collective focusing on recurrence rate and 30-day postoperative outcomes. In general, the authors of this study follow a tailored, step-up approach for complex midline incisional hernia repair as previously described by Niebuhr et al. [18]. The goal of mesh reinforcement in sublay position and restoration of the myofascial ring by reconstruction of the white line is to be achieved by starting with less invasive and technically demanding techniques (BTA, Rives-Stoppa and IFT) and ultimately leads to more challenging techniques including component separation and bridging. However, especially TAR is used if additional lateral defects exist or a wider mesh overlay is necessary.

### BTA and IFT

BTA for chemical relaxation of the lateral abdominal wall has become quite popular in recent years. Several studies have shown very positive results, although there are no randomized controlled trials available yet [26–28]. According to recent studies, reduction of hernia width of about 5 cm and myofascial advancement of approx. 4 cm is achievable per side [27, 29]. In our cohort, 87% of all cases were pretreated with BTA. Four of five hernia centers participating in this study use BTA routinely for midline hernias with a transverse diameter above 8–10 cm. In our study, intraoperative findings on myofascial elongation in each case were not recorded. Therefore, the influence of BTA cannot be differentiated. Nevertheless, all participating hernia centers did not utilize IFT if low-tension closure of the anterior rectus sheaths were deemed possible by the responsible surgeon. Interestingly, BTA had no significant influence on the closure rate of the anterior rectus sheaths.

### Fascial closure and bridging

Bridging of the anterior rectus sheath should absolutely be avoided as it significantly increases the risk of recurrence [30]. We had a primary closure rate for the anterior rectus sheaths of 94% in our cohort which is in line with results from high-volume single center data (92%) and slightly lower than findings from a systematic review (98%) and newly published data from Ghent (98.2%) regarding TAR [31–33]. However, apart from data from Novitsky, which were included in the systematic review, and Zolin, the defect width was considerably smaller in the other studies. Recently, a cadaveric model has shown that IFT provides comparable medialisation of the anterior rectus sheath compared to established component separation techniques [34]. If low-tension closure of the anterior rectus sheaths could not be achieved after IFT, the peritoneal flap technique or mesh for bridging was used [19]. Interestingly, performing a TAR has not had a significant impact on the anterior sheath closure rate in our data, which is in line with findings that the TAR is less effective than anterior CS in terms of anterior sheaths approximation [35, 36]. In conclusion, IFT shows similar fascial closure rates compared to component separation techniques but avoids extensive preparation.

Concerning the posterior rectus sheaths, cadaveric studies have shown superiority of the TAR over the anterior component separation concerning medialization [35, 36]. However, there is an ongoing debate about whether direct closure of the posterior sheaths is also necessary. To our knowledge, there are no studies showing an advantage of direct closure of the posterior sheath over bridging. Additionally, an older biomechanical analysis found that the posterior rectus sheaths are significantly less resistant to bursting than the anterior rectus sheaths [37]. In our opinion, utilising retrorectus mesh augmentation using a synthetic mesh always needs to be combined with a closed layer between the abdominal contents and the mesh to avoid adhesions and potential bowel erosion. If direct closure of the posterior sheaths was not possible, we performed the peritoneal flap technique (some call it closure, some call it bridging), which (as a standalone technique) has shown favourable outcomes regarding SSO and recurrence rates [19, 38].

### Mesh overlap

The use of IFT in abdominal wall reconstruction of midline incisional hernias is normally combined with a retrorectus dissection as described by Rives-Stoppa [39, 40]. The resulting space is used for sublay mesh augmentation. Therefore, the median mesh width appears relatively low with 20 cm in our cohort. Since the first description of posterior component separation (PCS) by Carbonell in 2008, a large mesh overlay has been advocated for the repair of complex abdominal wall hernias. Interestingly, Carbonell only mentioned the possibility of using a larger mesh if a PCS is carried out without providing evidence that this is truly necessary [41]. To our knowledge, there is no study to date that shows a reduction in the recurrence rate with the insertion of a larger mesh in open ventral hernia repair [42]. Like other authors, we believe that restoring and closing the myofascial ring of the abdominal wall is a key step in ventral hernia repair, perhaps more than having a large mesh overlap [38]. However, performing PCS and especially TAR results in an extended plane up to the spine which allows the placement of a large mesh. This is undoubtedly necessary if an additional lateral defect is present. In our clinical experience, once this space has been created, a wider mesh is usually inserted regardless of the circumstances. Also in our cohort, when an additional TAR was performed, a significantly wider mesh was used (*p* = 0.012; α = 0.05). Therefore, the defect-to-mesh ratio was higher if an additional TAR was performed (1.97 vs. 2.51 respectively). In addition, some authors of this study performed a TAR in selected cases and inserted a larger mesh to avoid possible hernia formation when a former stoma site was present. Interesting results regarding mesh strength and dislocation were reported by the group of Kallinowski from Heidelberg. They developed a model called dynamic intermittent strain (DIS) to simulate coughing by applying short impacts of 250 mmHg on a piece of the mesh using a pig tissue model [43]. Following the DIS concept, to bridge a gap of 5 cm in diameter, an overlap of 5 cm in all directions is sufficient if a DIS class A mesh is used. If the peritoneum/posterior sheath is closed, no mesh dislocation is seen. The overlap could even be reduced to 2.5 cm [44]. The Dynamesh Cicat^®^ mesh (FEG Textiltechnik, Aachen, Germany) which was used in 92.9% of all cases in our cohort was defined as a DIS class A mesh. We therefore consider that an excessive overlap is not mandatory and a TAR is not needed if the lateral abdominal wall is left intact and the midline restored. In our study, the recurrence rate was not different in the TAR group (1/28) with a larger overlap and the IFT group (1/72) with Rives Stoppa only.

### Postoperative complications

For data collection, we conducted a retrospective analysis of the 30-day postoperative outcome. We found an overall SSO rate of 33% and an overall SSI rate of 9%. The majority of these SSOs were seromas (54.5%). Remarkably, both the SSO rate and the seroma rate, was significantly higher if an additional TAR was performed (26.4% vs. 50% and 12.5% vs. 32.1%; *p* = 0.024 and *p* = 0.008; α = 0.05). 16 patients needed an intervention either as aspiration and drain of seroma/hematoma or as wound revision. And again, the rate of intervention was higher if an additional TAR was performed (25% vs. 12.5% respectively). In general, the SSO rate of 33% appears to be higher than previously published data for IFT [16, 17]. However, more recent data have shown similar results [34]. One explanation is the relatively obese patient cohort with a mean BMI of 31.3 kg/m². A retrospective review of the ACHQC has shown that the risk for complications significantly increases when the BMI exceeds 30 kg/m² [45]. Furthermore, 54.5% of all SSOs in our patient cohort were seromas. Since all participating hospitals are specialised hernia centers, patients are closely followed up by out-patient appointments within 30 days after surgery including physical examinations and routine ultrasound. Hence, even small and clinically silent seromas were meticulously recorded. Some authors suggest that seromas that do not cause any symptoms or lead to further complications should possibly not be considered complications or SSOs at all [46, 47]. In addition, seroma formation can be found in up to 100% of cases after laparoscopic IPOM for incisional hernia repair using ultrasound examination [48]. Morales-Conde has even suggested a classification solely for seromas and categorising them into non clinical seroma, incident or complication [49]. Looking at our data, the overall rate of SSO would decrease to 20% if only the seromas requiring intervention were counted as SSO. In addition, there was not standard for the treatment of seromas and the thresholds for seroma aspiration were different in the participating hernia centres.

Regarding open TAR, a recent systematic review and meta-analysis have found an overall complication rate of 33.34%, an intervention rate of 9.82%, and a SSI rate of 9.13% in twenty-two studies [50]. Oprea et al. found an overall wound morbidity rate of 28.63% and a SSI rate of 10.6% in a systematic review of anterior and posterior component separation [51]. A systematic review for giant incisional hernias (as defined in a hernia width above 10 cm) found a SSO rate of 21.4% for open anterior CS and 23.7% for TAR respectively [5]. Apart from the latter, the SSO and SSI rates are consistent with our results for IFT. Additionally, intervention rates of 9.82% and 8.36 have been reported for TAR, which is slightly lower than the overall intervention rate in our cohort [50, 51]. However, it is noteworthy that all systematic reviews had heterogeneity regarding the definition of SSOs and intervention in the reviewed studies which could lead to different rates. Interestingly, like the SSO rate, the SSI rate was higher if an additional TAR was performed in our cohort but not statistically significant (10.7% vs. 8.3% respectively). In our cohort, when IFT was combined with TAR, the risk for wound morbidity increased. This is in line with results from a propensity-scored matched study by Marturano comparing BTA and CS showing no difference in fascial closure rates but higher incidence of SSO when performing a CS [52].

### Dynamic ultrasound for follow-up

Different tools and measurements are used to detect hernias or assess possible recurrence after hernia repair. Clinical examination is the easiest and probably oldest method to diagnose a hernia/recurrent hernia. Regarding recurrences, it has a reported sensitivity of 75% and specificity of 90% [7]. The most common diagnostic tools are probably CT scans or MRIs. Interestingly, even though most authors and clinicians are using CT scans for preoperative planning in ventral/incisional hernia repair the sensitivity and specificity are on the same level as clinical examination (79% and 94%, respectively) [53]. In the authors’ practice ultrasound has a great value and is used daily. It was shown that dynamic abdominal wall ultrasound has a sensitivity of 98% and specificity of 88% for incisional hernias and is therefore not inferior to a CT scan [7]. Since the EHS either recommends ultrasound or a CT scan for follow-up, we decided to combine physical examination and dynamic abdominal wall ultrasound (DAWUS) using a specially designed protocol (supplementary data) [54]. Hence, we avoided exposure to radiation. A recurrence was detected in two patients. They received a CT scan afterwards, which confirmed the recurrence. Both patients were treated surgically.

### Recurrence rate

Utilising physical examination and a standardized ultrasound protocol, we found a recurrence rate of 2% at a mean follow-up time of 19.6 ± 10.7 months. Novitsky found a recurrence rate of 3.7% after a mean follow-up of 31.5 months in his single center TAR cohort, which is in line with a systematic review by Wegdam et al. from 2019 and Oprea et al. from 2023 (4% after 2 years and 6.11% after minimum 1-year follow-up respectively) [31, 51, 55]. However, the group of Rosen recently found a composite recurrence rate of 26% for TAR after a median follow-up of 2 years in their patient cohort [33]. It is noteworthy that in our cohort a recurrence occurred in two cases, whereas one patient was initially treated with an additional TAR and the other had IFT and retrorectus dissection.

### Quality of life

Surgeons often focus on clinically measurable outcomes like SSO and recurrence rates. For patients, an even greater significance is quality of life (QOL), including mental health and other areas of well-being [56–58]. Several attempts have been made to measure QOL, especially concerning the function of the abdominal wall in hernia patients with the Carolina Comfort Scale (CCS) and HerQles being the most sophisticated and established [59, 60]. This is the first study to investigate QOL in ventral hernia repair using IFT. We used the HerQles questionnaire as CCS focuses more on mesh sensation. In our cohort, we found a mean HerQles summary score of 68.5 ranging from 0 (worst) to 100 (best). This is compatible with a large survey of 1817 patients with ventral hernia repair from the Abdominal Core Health Quality Collaborative (ACHQC) that showed a mean HerQles score of 74.9 1-year postoperatively. However, it should be noted, that all types of surgical approaches were included (open, laparoscopically, robotically) in the ACHQC analysis. Regarding TAR, other retrospective analyses have shown higher HerQles summary scores (82 and 91.8 respectively) [61, 62]. However, these studies presented single-surgeon resp. single-center data with a short follow-up of 6 months and one year respectively. A limitation of our study is the lack of a preoperative assessment of QOL which unfortunately does not allow any conclusions to be drawn about postoperative improvement.

### Study limitations

All patients in our cohort answered the HerQles questionnaire only postoperatively during follow-up but not before surgery. It is therefore not possible to compare QOL pre-and post-operatively. Furthermore, our standardised dynamic ultrasound, although seeming feasible for follow-up, needs further validation by comparing the results with CT scan or MRI. Additionally, since hernia recurrences can occur even after many years, a longer follow-up period for patients treated with IFT is needed. Finally, comparative studies with other techniques like component separation are needed to determine which technique is most suitable for the surgeon’s intended goal and for each patient individually.

## Conclusion

In this multicenter study, we present outcomes for IFT in complex hernia repair. In this large series with wide defects and an obese patient population, we show that very high direct closure rates for the anterior sheath with low wound infection rates and a very low recurrence rate can be achieved with the use of IFT, thus making IFT a simple but widely usable tool in the armamentarium for complex abdominal wall surgery. However, every patient who presents with a complex ventral hernia needs a tailored approach following an algorithm that includes BTA, Rives-Stoppa preparation, peritoneal flap, IFT and component separation or bridging if needed. At present, it remains unclear which technique has the greatest benefit for the respective indication and additional studies are necessary. For determination of recurrences, we developed a protocol for standardised, dynamic abdominal wall ultrasound which needs further validation against CT/MRI. Future studies should focus on comparative trials comparing the closure rate and recurrence rate of IFT and component separation techniques. The question of the extent of mesh overlap in ventral hernia repair should be addressed additionally, especially considering the closure of the anterior rectus sheath.
